# Unravelling mechanisms of drought tolerance in a soybean cultivar (Daewonkong roots): insights into integrative transcriptomic and metabolite analyses

**DOI:** 10.1186/s12870-026-08144-2

**Published:** 2026-01-15

**Authors:** Yo-Han Yoo, Jinsil Yeo, Doheon Choi, Ye-Jin Son, Hyangyeon Jeong, Sangjun Park, Yeon Ju An, Girim Park, Eunsoo Lee, Mi-Suk Seo, Ju Sung Im, Soo-Kwon Park, Ki-Hong Jung, Woo-Jong Hong

**Affiliations:** 1 Department of Upland Crop Sciences, Rural Development Administration, Upland Crop Breeding Division, National Institute of Crop and Food Science, Miryang, 50424 Republic of Korea; 2 Department of Smart Farm Science, Kyung Hee University, Yongin, 17104 Republic of Korea; 3 Graduate School of Green Bio-Science & Crop Biotech Institute, Kyung Hee University, Yongin, 17104 Republic of Korea; 4 Plant Biomaterials and Biotechnology Division, Department of Agricultural Biology, National Institute of Agricultural Sciences, 370, Nongsaengmyeong-Ro, Deokjin-Gu, Jeonju City, 54874 Jeonbuk-Do Korea

**Keywords:** Soybean (*Glycine max*), Drought stress, Transcriptome analysis, Isoflavones, Antioxidant activity, Stress-responsive genes

## Abstract

**Background:**

Soybean (*Glycine max* L.), a major food crop in Korea, is highly vulnerable to drought, particularly under rain-fed cultivation. Although several transcriptomic studies have examined drought-responsive pathways in soybean leaves, research on root-specific responses and their association with isoflavone-mediated antioxidant defense remains limited. The Korean cultivar Daewonkong, which is widely cultivated but sensitive to drought, presents a useful candidate for investigating the molecular and metabolic mechanisms of stress susceptibility.

**Results:**

RNA sequencing of Daewonkong roots under controlled and drought-stressed conditions identified 1,348 upregulated and 2,835 downregulated genes. Kyoto Encyclopedia of Genes and Genomes and MapMan analyses revealed enrichment of galactose, nitrogen, and glutathione metabolism among the upregulated genes, whereas cell wall, lipid, phenylpropanoid, and isoflavonoid biosynthesis were strongly repressed, suggesting a metabolic shift from growth-related processes to stress acclimation. When comparing the drought-sensitive Daewonkong cultivar with the drought-tolerant cultivar PI 471938, clear phenotypic and metabolic differences were observed. PI 471938 displayed a substantially higher survival rate after recovery from drought-induced stress and accumulated 2.5-fold greater levels of total isoflavones. Concurrently, this cultivar exhibited significantly enhanced antioxidant capacity, with higher polyphenol content and stronger radical scavenging activity [2,2′-Azino-bis(3-ethylbenzothiazoline-6-sulfonic acid) (ABTS) and 2,2-Diphenyl-1-picrylhydrazyl (DPPH)] than Daewonkong. Furthermore, several drought-responsive genes, including *GmMYB14*, *GmNFYA13*, *GmWRKY12*, and *GmFAD3A*, were expressed at high levels in PI 471938, consistent with their roles in oxidative stress mitigation, membrane stability, and transcriptional regulation.

**Conclusions:**

Our findings demonstrate that drought tolerance in soybean is associated with enhanced antioxidant activity, increased isoflavone accumulation, and the coordinated induction of stress-responsive genes. These results provide molecular insights into soybean’s drought adaptation, establishing a foundation for breeding strategies to improve stress resilience.

**Supplementary Information:**

The online version contains supplementary material available at 10.1186/s12870-026-08144-2.

## Background

Soybean (*Glycine max* L.) is a globally essential crop cultivated for both vegetable oils and dietary proteins. In 2020, the global soybean production exceeded 353 million tons, with Brazil and the United States together accounting for nearly two-thirds of the total output [1]. Most soybean cultivation occurs under rain-fed conditions, making its yield particularly vulnerable to water deficits and climate variability [2]. Drought stress is found to be a major constraint on soybean productivity, as it hampers photosynthetic efficiency, disrupts assimilate allocation, and ultimately lowers yield [3]. Physiological responses to drought include reduced leaf water potential, loss of membrane integrity, and the accumulation of reactive oxygen species (ROS), which impair cellular function [4]. Early vegetative drought can also alter canopy temperature and root-to-shoot ratio, increasing root biomass but reducing shoot weight, thereby affecting biomass accumulation and yield stability [2]. Therefore, improving drought tolerance in soybeans is critical for sustaining production and ensuring food security under increasingly variable climatic conditions.

RNA sequencing (RNA-seq) is a powerful, high-throughput approach for genome-wide quantification of gene expression, enabling precise identification of stress-responsive genes across developmental stages. Its high sensitivity and broad dynamic range render it suitable for dissecting complex responses such as those induced by drought stress [5]. In soybean, transcriptomic profiling of leaves from drought-tolerant SS2-2 and drought-sensitive Taekwang cultivars under control and drought conditions revealed differential expression of genes involved in signaling, lipid metabolism, phosphorylation, transcriptional regulation (e.g. WRKYs, NACs), Mitogen-activated protein kinase (MAPK) and Ca^2^⁺ signaling, ROS scavenging, and Nucleotide-binding site-leucine-rich repeat (NBS-LRR) pathways [6]. Another study integrating RNA-seq and metabolomics in soybean seedlings demonstrated that drought substantially altered both transcript and metabolite profiles in the leaves, with key changes witnessed in isoflavone biosynthesis and the Tricarboxylic acid (TCA) cycle [7]. However, most RNA-seq studies regarding drought stress in soybeans have focused on leaf tissues, while roots assessment remain relatively limited [8].

High-performance liquid chromatography (HPLC) is widely used in plant metabolite profiling due to its high precision, reproducibility, and ability to resolve complex isoflavone mixtures in soybean tissues [9]. Reversed-phase C18 columns coupled with UV or diode array detection (DAD) allow simultaneous quantification of both aglycones (e.g., daidzein and genistein) and glycosides (e.g., daidzin, genistin, and malonyldaidzin) in a single run [10]. Sun et al. developed a rapid HPLC–UV method to measure 12 soybean seed isoflavones, including daidzin, malonyldaidzin, and genistein, with high linearity, repeatability, and suitability for breeding applications [10]. In research involving drought-induced stress, Qin et al. employed HPLC to quantify isoflavones in leaves and roots of two soybean cultivars (JP-6 and JP-16), revealing higher root isoflavone accumulation in the drought-tolerant cultivar [11]. A recent comparative study demonstrated that heterogeneous drought conditions elicited increased isoflavone levels in soybean seedlings (assessed using HPLC–DAD), suggesting adaptive metabolic responses [12]. These studies have consistently reported that glycoside and aglycone isoflavone levels correlate positively with antioxidant enzyme activity and drought tolerance indicators, reinforcing their potential as physiological markers.

Daewonkong is one of the most commonly cultivated soybean (*Glycine max* L.) varieties in South Korea, and was commercially released in 1997 due to its agronomic stability and suitability to mechanized farming systems. It maintains a stable yield performance and phenological traits under variable temperature conditions, demonstrating its adaptability to temperate regions [13]. However, Daewonkong is particularly sensitive to drought stress, exhibiting more pronounced yield loss than other Korean cultivars. Under water-deficit conditions, it displays greater reduction in pod number per plant and 100-seed weight compared to Daepung-2ho and Pungsannamul, resulting in significant yield instability [14].

In the present study, Daewonkong was subjected to controlled drought treatment to evaluate its transcriptomic responses in root tissues and explore the underlying mechanism of drought tolerance. Differentially expressed genes (DEGs) were identified using RNA-seq, and functional enrichment analyses were conducted using Kyoto Encyclopedia of Genes and Genomes (KEGG) enrichment and MapMan visualization. Furthermore, the phenotypic responses of Daewonkong were compared with those of a drought-tolerant cultivar (PI 471938), and the isoflavone content was quantified to explore potential metabolic adjustments under stress. These analyses aimed to provide a comprehensive understanding of the molecular and physiological mechanisms underlying drought responses.

## Results

### Physiological responses of soybean to drought stress

**Fig. 1:**
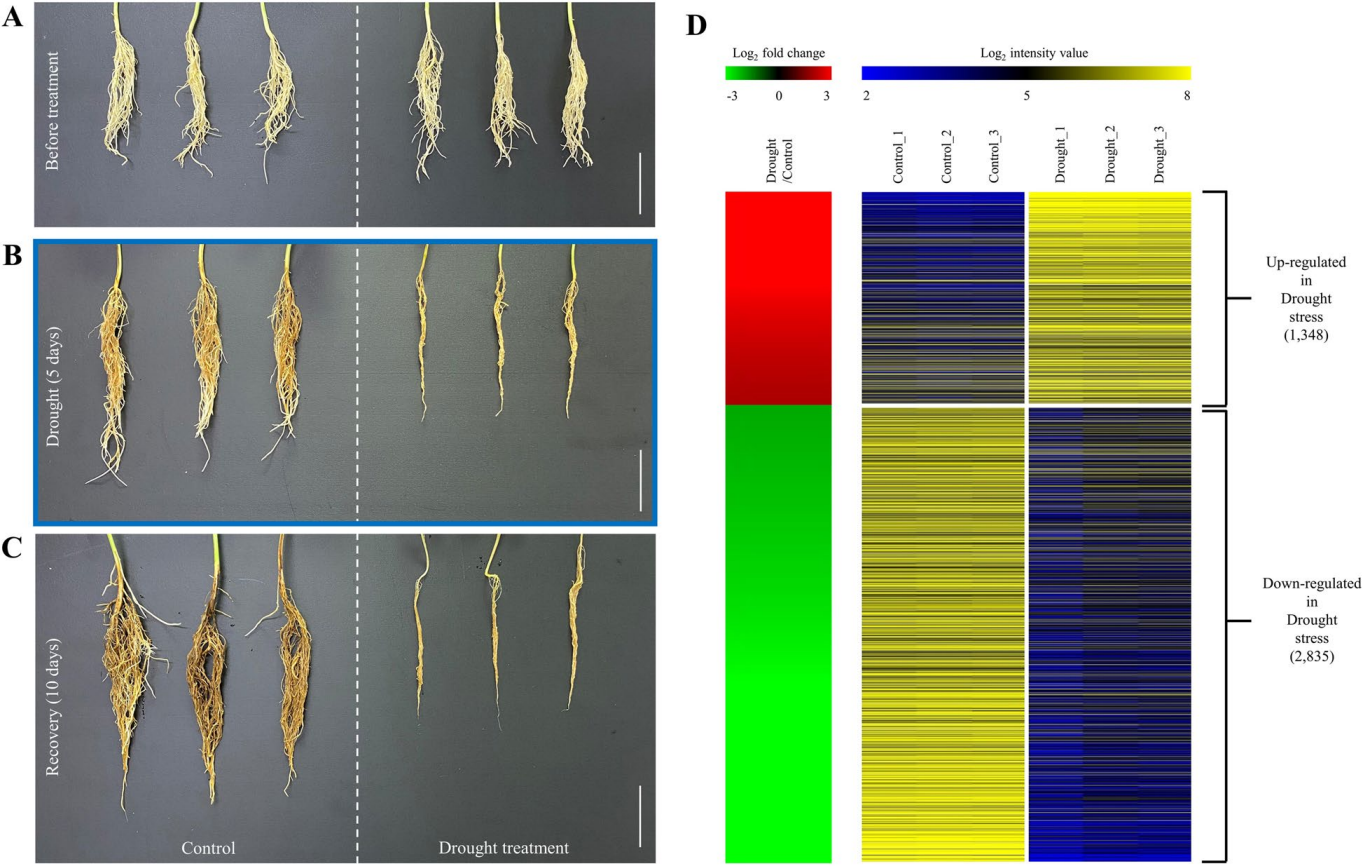
Physiological responses of soybean roots to drought stress and transcriptome profiling of differentially expressed genes (DEGs). **A**–**C** Root phenotypes of Daewonkong seedlings at the V2 stage before drought treatment (**A**), after 5 d of drought stress (**B**), and after 10 d of recovery (**C**). Scale bar = 5 cm. *N* = 3. **D** Heatmap of DEGs in roots under drought stress compared to the control plants. RNA-seq analysis was performed using a threshold of *Padj* < 0.01, and |log₂ fold change|≥ 2, identifying 4,183 DEGs. In the left panel, red indicates upregulated genes and green indicates downregulated genes in drought-stressed roots relative to the controls. The right panel shows the normalized read count values across replicates, with blue indicating the lowest expression and yellow indicating the highest expression. Detailed DEG information is provided in Additional File 1 of Table S1. DEG: differentially expressed genes

To assess drought stress responses, soybean seedlings (V2 stage) grown for two weeks under controlled conditions were exposed to drought for five days, followed by rewatering for ten days (Fig. 1A–C). The V2 stage corresponds to the onset of active root development and nitrogen fixation, accompanied by fully expanded trifoliolate leaves. Because soybean seedlings exhibit high physiological sensitivity to soil moisture deficit at this stage, it is widely used as a standard time point for seedling drought assays, and we therefore adopted the V2 stage for our experiment [15, 16]. The root phenotypes were assessed at three time points: before treatment, after drought exposure, and after recovery. Control plants displayed continuous root elongation, whereas drought-stressed plants showed growth arrest during treatment and failed to resume growth after rewatering (Fig. 1A–C). Leaf morphology gradually deteriorated during the drought period, and by day five, most of the leaves had wilted, turning pale green. During the recovery phase, the leaves did not regain their original appearance, showing progressive yellowing and abscission; however, a few plants exhibited partial recovery with newly emerging healthy leaves. These observations suggest that drought tolerance should be evaluated immediately after the stress treatment and during the recovery phase.

### Identification of DEGs in response to drought stress

RNA-Seq was performed on root samples from control plants and plants subjected to drought stress for five days. In total, 1,348 genes were significantly upregulated and 2,835 genes were significantly downregulated in drought-stressed roots compared with controls (*Padj* < 0.01, |log₂ fold change|≥ 2; Fig. 1D, Additional file 1: Table S1). The number of downregulated genes was approximately 2.1-fold greater than the number of upregulated genes. A heat-map was generated to illustrate the expression profiles of 4,183 DEGs, including both upregulated and downregulated genes (Fig. 1D). This heat-map was designed from log₂ fold-change values between control and drought-stressed roots, together with log₂ expression intensities from all replicates.

### Identification of enriched biological processes in soybean roots under drought stress

**Fig. 2:**
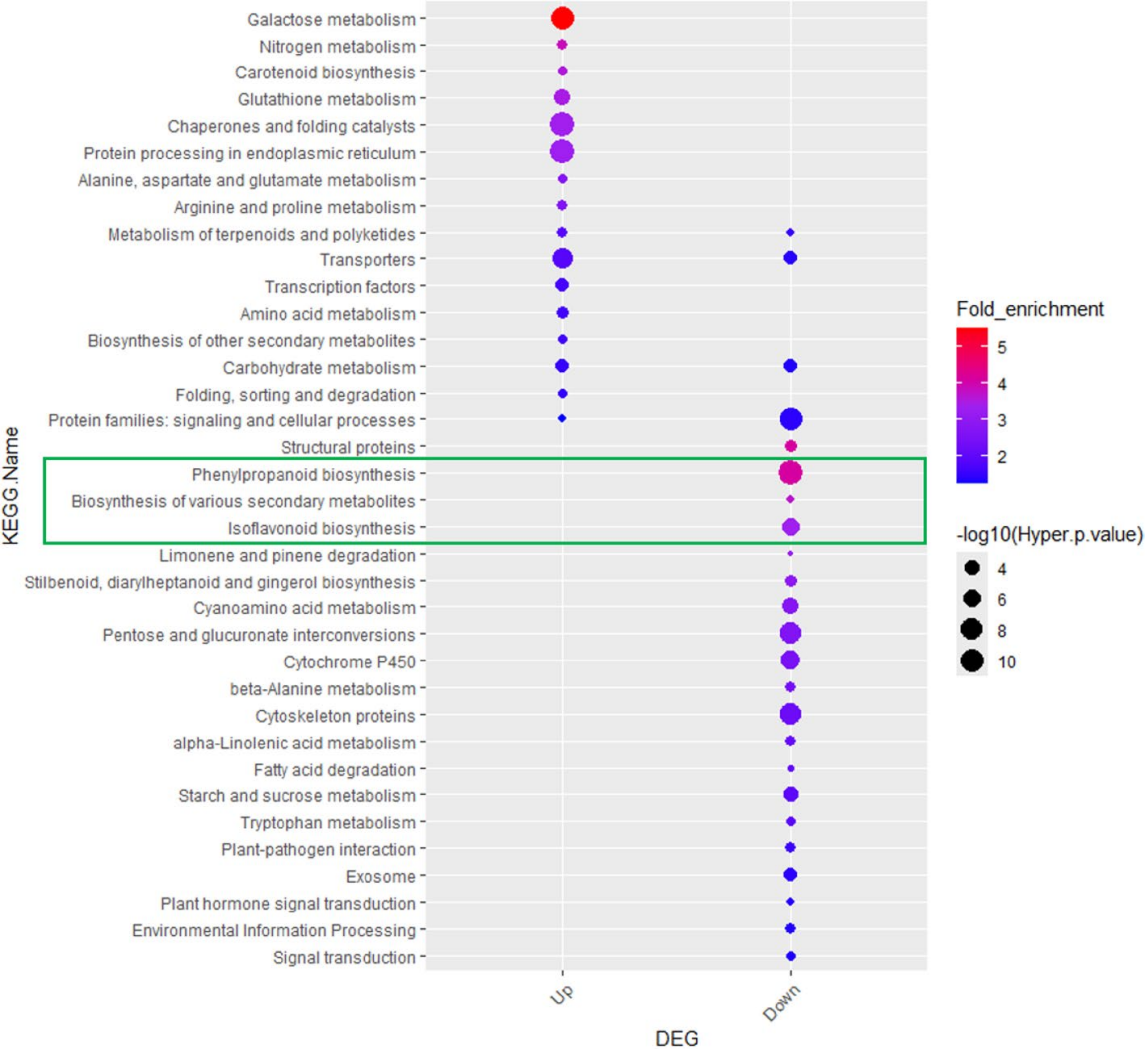
KEGG enrichment analysis of DEGs under drought stress. KEGG pathway enrichment was performed for genes that were upregulated or downregulated in Daewonkong roots in response to drought stress. Sixteen pathways were significantly overrepresented among upregulated genes, whereas 24 pathways were significantly enriched among downregulated genes (*p* < 0.05, fold enrichment > log₂1). The detailed KEGG assignments are provided in Additional File 2 (Table S2). KEGG: Kyoto Encyclopedia of Genes and Genomes

To elaborate on the functions of 1,348 upregulated and 2,835 downregulated genes in soybean roots under drought stress conditions, KEGG enrichment analysis was performed. Sixteen terms were significantly overrepresented among the upregulated genes, while 24 terms were enriched among the downregulated genes (enrichment factor (eF) > 1, *p-value* < 0.05). In the upregulated gene set, metabolism-related pathways such as galactose metabolism (eF = 5.50; *p-value* = 6.37E-10), nitrogen metabolism (3.87; 0.001), and glutathione metabolism (3.47; 4.97E-06) were highly enriched (Fig. 2, Additional file 2: Table S2). Conversely, in the downregulated gene set, biosynthesis-related pathways, including phenylpropanoid biosynthesis (4.12; 0.000), biosynthesis of various secondary metabolites (3.70; 0.005), and isoflavonoid biosynthesis (3.36; 3.04E-06), were predominantly represented (Fig. 2, green box).

### Functional classification of drought stress-related genes in soybean roots

**Fig. 3:**
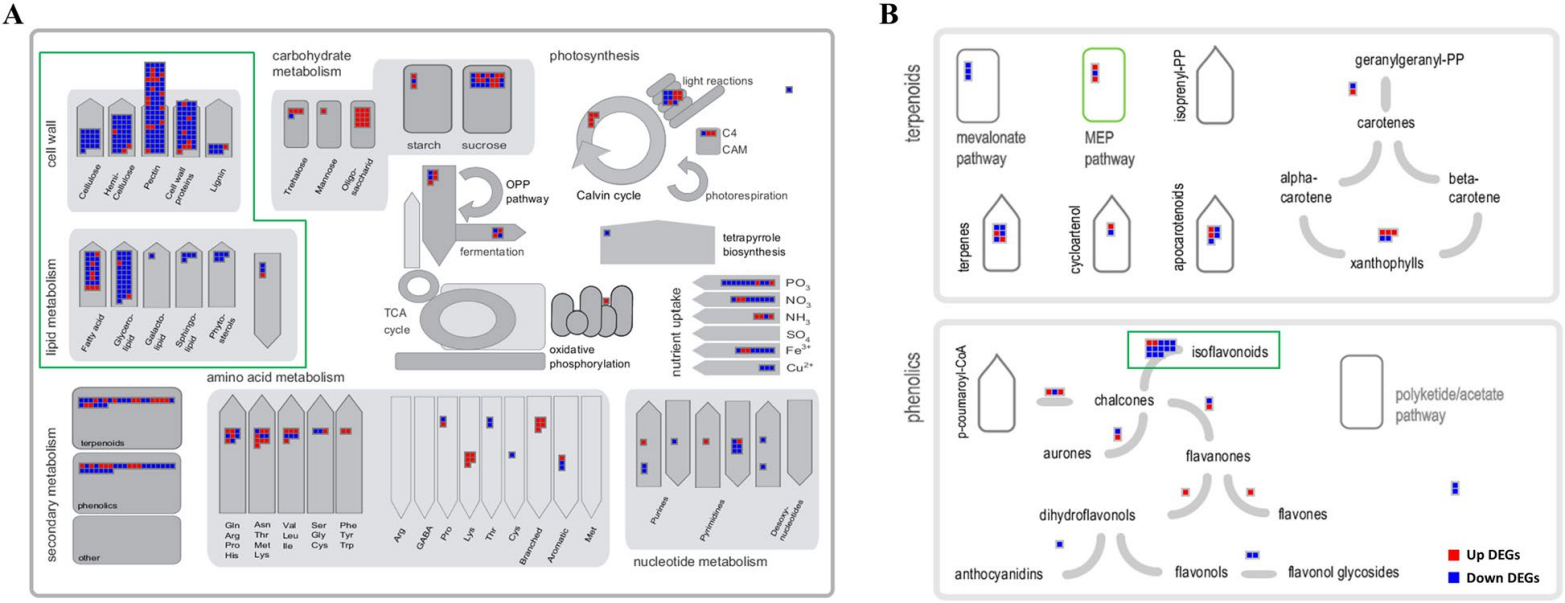
MapMan analysis of DEGs under drought stress. MapMan overview shows the distribution of drought-responsive genes in (**A**) primary and (**B**) secondary metabolism. Red boxes indicate upregulated genes, and blue boxes indicate downregulated genes. Detailed information on the MapMan assignments is provided in Additional File 5 and Table S5

MapMan is a visualization tool that enables functional classification and mapping of genes onto biological pathways. In this study, 1,348 upregulated and 2,835 downregulated genes were identified using MapMan (Fig. 3). Upregulated genes are shown as red boxes while downregulated genes are shown as blue boxes. In the metabolism overview, many downregulated genes were identified in categories related to cell wall and lipid metabolism (Fig. 3A, green box). Consistent with the KEGG enrichment analysis, numerous downregulated genes were also observed in secondary metabolism, particularly in pathways associated with phenolics and terpenoids (Fig. 3B).

A substantial number of downregulated genes identified under drought stress in soybean roots were associated with cell wall metabolism (e.g., cellulose, hemicellulose, pectin, and cell wall proteins) and lipid metabolism (e.g., fatty acids, glycerolipids, phytosterols, and sphingolipids) (Fig. 3A).

### Differential isoflavone and antioxidant responses in Daewonkong and PI 471938 under drought stress

KEGG enrichment and MapMan analyses revealed that a substantial number of downregulated genes in drought-stressed Daewonkong roots were associated with secondary metabolism, including pathways related to isoflavonoid biosynthesis (Fig. 2, Fig. 3B, green box).

**Fig. 4:**
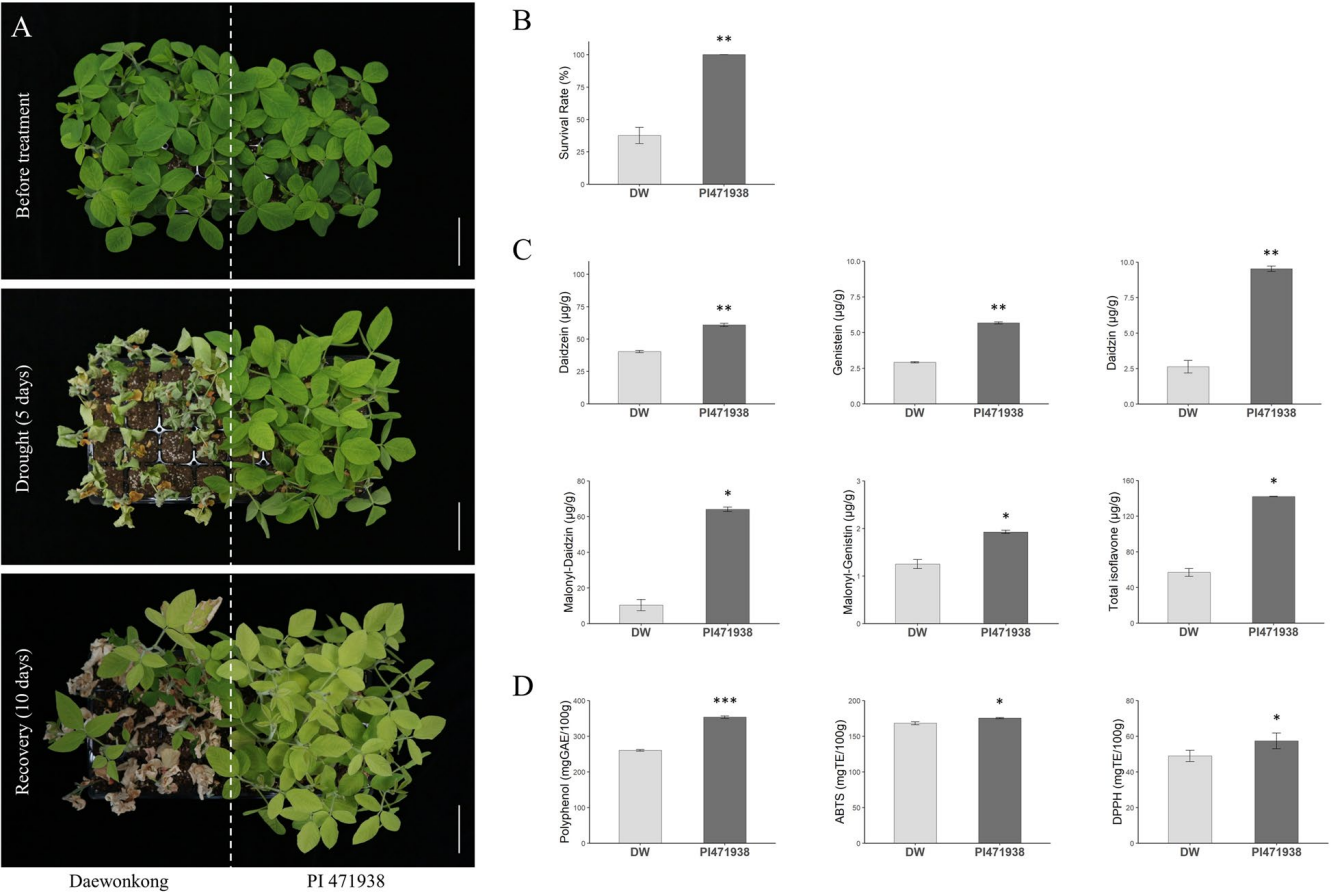
Isoflavone accumulation and antioxidant responses in Daewonkong and PI 471938 under drought stress. **A** Seedling phenotypes after 5 days of drought stress and 10 days of recovery. Scale bar = 5 cm. *N* = 3. **B** Survival rate was 37.5% in Daewonkong, whereas all PI 471938 plants survived. **C** Isoflavone profiling in roots showed significantly higher levels of key compounds and total isoflavones in PI 471938 compared with Daewonkong. **D** Antioxidant assays revealed higher total polyphenols, 2,2′-Azino-bis(3-ethylbenzothiazoline-6-sulfonic acid (ABTS)), and2,2-Diphenyl-1-picrylhydrazyl (DPPH) activities in PI 471938. *p < 0.05; **p < 0.01, ***p < 0.001

To compare drought tolerance phenotypes between Daewonkong and PI 471938, seedlings at the V2 stage were subjected to drought stress for 5 days, followed by a 10-day recovery period (Fig. 4A). During the recovery phase, Daewonkong exhibited a survival rate of 37.5%, whereas all PI 471938 plants survived, indicating a substantially higher drought tolerance (Fig. 4B). Root samples were collected after 5 days of drought treatment to quantify 12 isoflavone compounds, including aglycones, glycosides, malonyl glycosides, and acetyl glycosides. Significant increases (*p* < 0.05) in daidzein and genistein (aglycones), daidzin (glycoside), and malonyl daidzin and malonyl genistin (malonyl glycosides) were detected in PI 471938 compared to Daewonkong (Fig. 4C). The total isoflavone compounds were also found to be 2.5-fold higher in PI 471938 (142.4 μg/g) than in Daewonkong (45.8 μg/g). Seven compounds with concentrations below 1 μg/g were excluded from the results.

Furthermore, to assess whether the increase in total isoflavone compounds influenced overall antioxidant capacity, total polyphenols, ABTS (2,2’-azino-bis), and DPPH (2,2-diphenyl-1-picrylhydrazyl) were quantified (Fig. 4D). Total polyphenol content was estimated to be significantly higher in PI 471938 [354.7 mg Gallic acid equivalents (GAE)/100 g] than in Daewonkong (259.4 mg GAE/100 g), representing a 36.7% increase (*p* = 6.17 × 10⁻⁶). ABTS activity, which reflects both water- and lipid-soluble antioxidant capacity, was 5% higher in PI 471938 (175.8 mg Trolox equivalents (TE)/100 g) than in Daewonkong (167.4 mg TE/100 g) (*p* = 0.0119). The DPPH activity, which is mainly responsive to phenolic antioxidants in organic solvents, was 22.6% higher in PI 471938 (58.3 mg TE/100 g) than in Daewonkong (47.6 mg TE/100 g) (*p* = 0.0231). These results indicated that the higher total isoflavone content in PI 471938 under drought stress may have contributed to its enhanced phenolic content and DPPH radical-scavenging activity.

### Differential expression of drought-related genes between Daewonkong and PI 471938

To further clarify the molecular mechanism underlying the drought-tolerant phenotype of PI 471938 compared to that of Daewonkong, we targeted previously reported drought-responsive genes with established functional roles in stress tolerance, rather than selecting genes directly from the RNA-seq-derived DEG dataset. In soybean, 31 genes have been reported to confer tolerance to drought or combined drought and salt stresses [17–19]. Using the same root samples subjected to five days of drought treatment at the V2 stage as those used for isoflavone and antioxidant analyses, we performed qRT-PCR(Quantitative real-time PCR) analysis.

**Fig. 5:**
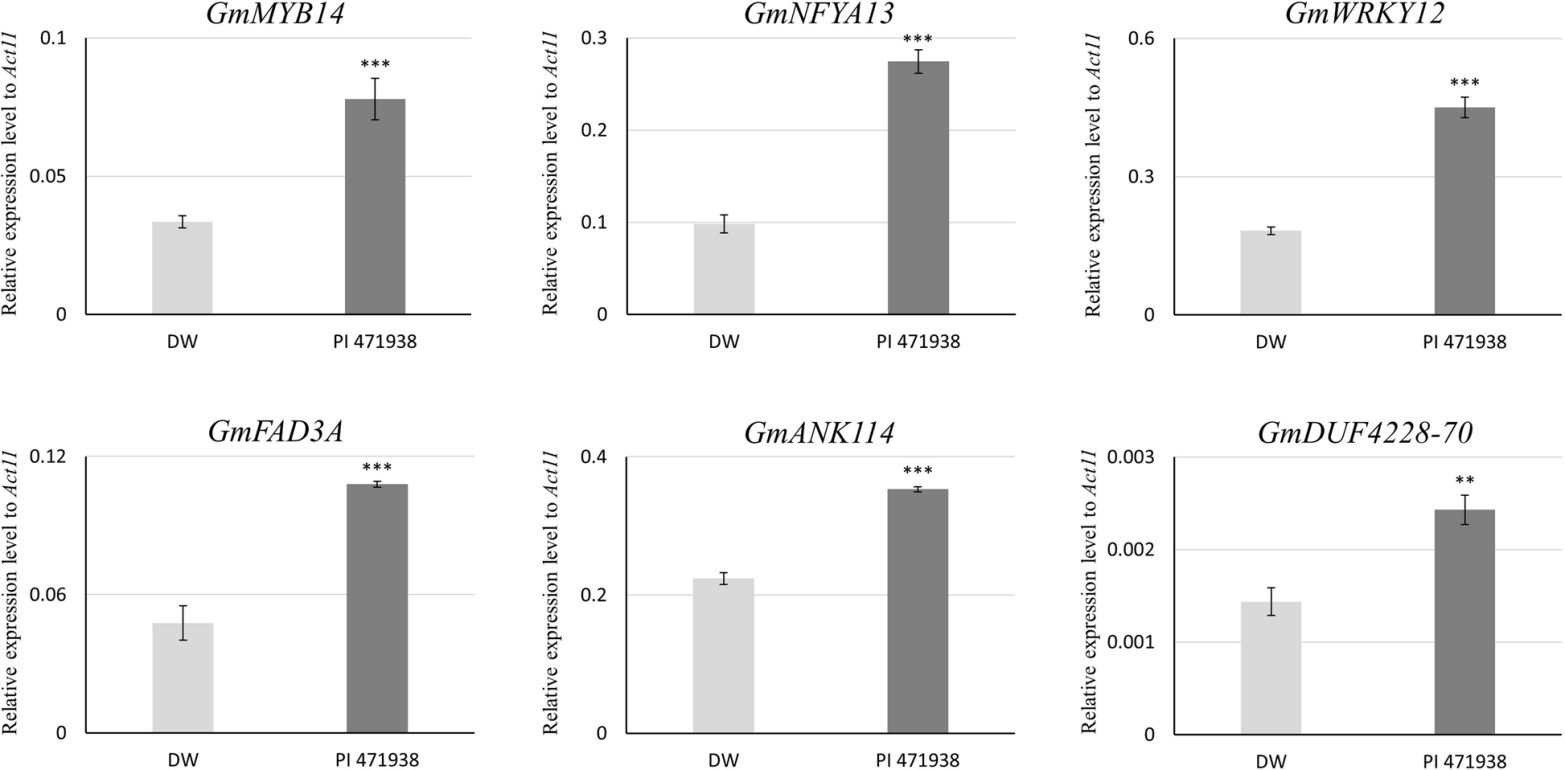
Real-time PCR validation of drought-responsive genes in Daewonkong and PI 471938 under drought stress. Expression levels of the six known drought-responsive genes were significantly higher in PI 471938 than in Daewonkong under drought stress. Transcript levels were determined using quantitative real-time PCR (qRT-PCR) and normalized to Act11 expression. Bars represent means ± SD of three biological replicates. DW: Daewonkong (light grey); PI 471938 (dark grey). **p < 0.01; ***p < 0.001. The primer sequences are listed in Additional file 4: Table S4

Among the 31 genes examined, four (*GmMYB14*, *GmNFYA13*, *GmWRKY12*, and *GmFAD3A*) showed more than a two-fold higher expression in PI 471938 than in Daewonkong (Fig. 5) [20–23]. In addition, two genes (*GmANK114* and *GmDUF4228-70*) were expressed at least 1.5-fold higher in PI 471938 compared with Daewonkong [24, 25].

## Discussion

This study investigated the root-specific transcriptional and metabolic reprogramming of the drought-sensitive soybean cultivar, Daewonkong, to elucidate the mechanisms underlying stress susceptibility. Our data revealed a clear dichotomy between primary and secondary metabolic responses, providing new insight into the constraints of drought adaptation in sensitive genotypes.

### Metabolic shift in drought-stressed Daewonkong

Consistent with our findings, previous studies have demonstrated that drought stress triggers metabolic pathways in soybean roots. For instance, raffinose family oligosaccharide biosynthesis genes, including galactinol synthase, are strongly upregulated in primary roots under water-deficit conditions, highlighting the activation of galactose metabolism [26]. A comprehensive transcriptome and translatome study also identified glutathione and galactose metabolisms as key enriched pathways in nodulated roots subjected to drought stress, reiterating their roles in antioxidant defense and osmoprotection [27]. In line with the transcriptomic evidence, a proteomic investigation further confirmed that drought stress significantly enhanced carbohydrate metabolism and glutathione-related antioxidant pathways in soybean roots [28]. Taken together, these studies provide strong evidence that the genes upregulated under drought stress are predominantly enriched in metabolic processes, particularly those involved in carbohydrate turnover and redox homeostasis.

In contrast to the upregulation of defense-related pathways, a substantial number of downregulated genes identified under drought stress in soybean roots were associated with cell wall metabolism, including cellulose, hemicellulose, pectin, and cell wall proteins (Fig. 3A). This repression is consistent with the general idea that plants reallocate metabolic resources away from growth-related processes to prioritize survival during water deficit. Previous studies have demonstrated that drought stress suppresses the expression of cellulose synthase and related genes, thereby reducing energy-demanding cell wall biosynthesis [29]. Similarly, hemicellulose and pectin metabolism were greatly downregulated under drought conditions, coinciding with reduced cell expansion [30]. In soybeans, the widespread downregulation of cell wall–associated genes in roots subjected to drought stress has also been reported [26]. Collectively, these findings suggest that suppression of cell wall biosynthesis in Daewonkong reflects a shift from growth to stress acclimation strategies.

Furthermore, a substantial proportion of the downregulated genes in drought-stressed soybean roots was associated with lipid metabolism, including fatty acids, glycerolipids, phytosterols, and sphingolipids (Fig. 3A). The inhibition of these pathways likely reflects a shift in resource allocation, as lipid biosynthesis is highly energy- and carbon-demanding. Under water deficit conditions, plants commonly suppress growth-related metabolic processes to prioritize survival. Aligning with this interpretation, drought stress has been shown to decrease the expression of MGDG synthase gene *AtMGD1* in Arabidopsis leaves [31], reduce the total levels of membrane lipids, including steryl esters, in oat roots [32], and decrease the accumulation of phosphoglycerolipids, particularly highly unsaturated species, in Festuca roots [33]. Collectively, these findings suggest that the widespread suppression of lipid biosynthesis in soybean roots represents a common metabolic strategy for conserving resources under drought stress.

### Genotype-specific suppression of secondary metabolism

Interestingly, KEGG enrichment and MapMan analyses revealed that a substantial number of downregulated genes were associated with secondary metabolism, particularly highlighting the suppression of phenylpropanoid and isoflavonoid biosynthesis pathways in the drought-stressed Daewonkong roots (Fig. 2, 3B, and Additional file 6: Figure S1). This behavior differs from previous reports showing that activation of these pathways contributes to drought tolerance; for example, Coutinho et al. [34] demonstrated that the root phenylpropanoid pathway is enhanced in drought-tolerant soybean genotypes, and Wang et al. [35] identified phenylpropanoid and isoflavonoid biosynthesis as key pathways underlying drought tolerance in soybean roots [14]. Therefore, the observed repression of these pathways is likely to reflect a genotype-specific response characteristic of the drought-sensitive Daewonkong. Based on this finding, we further investigated whether the regulation of secondary metabolite pathways differs from that in a drought-tolerant cultivar, PI 471938, with particular emphasis on isoflavonoid biosynthesis.

### Enhanced secondary metabolism and drought tolerance in PI 471938

To validate the critical role of secondary metabolism, we compared the responses of Daewonkong with the drought-tolerant cultivar PI 471938. PI 471938 exhibited a distinct metabolic profile characterized by a 2.5-fold higher accumulation of total isoflavones and significantly enhanced antioxidant capacity (ABTS and DPPH) under drought stress compared to Daewonkong.

Since isoflavone profiling was performed only under drought conditions, it is not possible to distinguish whether the higher isoflavone levels in PI 471938 arise from constitutively elevated basal levels or stronger drought-induced accumulation. Nevertheless, the higher isoflavone levels observed under drought stress are strongly associated with the superior drought tolerance of PI 471938 compared with the sensitive cultivar Dawonkong.

Furthermore, key stress-responsive genes, including *GmMYB14, GmNFYA13, GmWRKY12, and GmFAD3A,* were highly expressed in PI 471938. These observations are consistent with previous functional studies demonstrating that the overexpression of these drought-related genes enhances stress tolerance through specific regulatory mechanisms. Specifically, *GmMYB14* improves drought resistance by activating flavonoid biosynthesis, thereby reducing oxidative damage [20], while *GmNFYA13* confers tolerance via the regulation of stress-responsive transcriptional networks [21]. Additionally, *GmWRKY12* enhances drought tolerance by modulating the abscisic acid (ABA)-responsive pathways and reducing ROS accumulation [22]. Similarly, *GmFAD3A* contributes to drought adaptation by maintaining membrane stability and enhancing osmoprotectant accumulation [23]. These findings highlight that the drought tolerance of PI 471938 is associated not only with enhanced isoflavone accumulation and antioxidant activity but also with the coordinated upregulation of multiple stress-responsive genes, support the view that robust secondary metabolism plays a critical role in drought adaptation.

## Conclusions

In this study, we performed RNA-seq analysis on the roots of the soybean cultivar Daewonkong under control and drought-stressed conditions and identified 4,183 DEGs associated with drought responses. Functional enrichment through KEGG and MapMan analyses highlighted pathways involved in stress adaptation, including antioxidant activity and secondary metabolite biosynthesis. After further comparison of Daewonkong with the drought-tolerant cultivar PI 471938, our results suggest that enhanced isoflavone accumulation, antioxidant capacity, and differential expression of known stress-responsive genes may contribute to the superior drought tolerance of PI 471938.

Nevertheless, a few limitations should be noted. Since metabolite profiling was conducted exclusively under drought conditions, further research is needed to elucidate whether the higher isoflavone levels in the tolerant cultivar are due to constitutively elevated basal levels or a stress-induced accumulation. Future studies involving functional validation of the identified candidate genes will be essential to confirm their direct contributions to drought resilience. Collectively, these findings offer new insights into the molecular basis of drought adaptation in soybean and suggest potential genetic targets for marker development and molecular breeding to enhance stress resilience.

## Materials and methods

### Plant growth conditions and drought stress treatment

Soybean (*Glycine max* L. Merr., cultivar Daewonkong) was grown in plastic seedling trays (BumNong, Gochang, Korea; 32 cells per tray, each cell containing 150 cm^3^ of soil; cell dimensions: 58 mm × 58 mm × 63 mm) filled with commercial potting soil (Baroker; Seoul, Korea; moisture content 40–60%, water-holding capacity 30–50%, and bulk density 0.15–0.25 Mg m⁻^3^) under controlled environmental conditions in a growth chamber (CONVIRON, Winnipeg, Canada; GEN 1000). The plants were maintained until they reached the V2 growth stage. Growth conditions were set to a 14 h light/10 h dark photoperiod, with daytime and nighttime temperatures of 28 °C and 24 °C, respectively, a relative humidity of 50%, and a light intensity of 620 μmol m⁻^2^ s⁻^1^. Drought stress was induced by withholding water for 5 days, followed by a 10-day rewatering period. Root samples were collected at three time points: prior to stress treatment, during drought stress, and after recovery.

### Transcriptomic analysis of root tissues

Root samples were harvested from control plants and plants subjected to five days of drought stress, with three biological replicates per condition. Each biological replicate consisted of pooled root tissues from three individual plants. Immediately after collection, the samples were flash-frozen in liquid nitrogen to preserve RNA integrity. Total RNA was extracted from the root tissues using the RNeasy Plant Mini Kit (Qiagen, Hilden, Germany). RNA quality was assessed using the Agilent 4200 TapeStation, and samples with RIN ≥ 7.0 and rRNA ratio ≥ 1.0 were used for further analysis.

Sequencing libraries were prepared and sequenced on the Illumina platform to generate 151 bp paired-end reads. Each sample yielded almost 60 million reads after trimming adaptor sequences and low-quality bases, which meets this recommended range (Additional file 3: Table S3). Raw reads were processed using Cutadapt (v3.5) with the parameters − a AGATCGGAAGAGC − A AGATCGGAAGAGC − q 20 − m 20, which removed adapter sequences, trimmed bases with Phred scores < 20, and discarded reads shorter than 20 bp. High-quality trimmed reads were then aligned to the *Glycine max* reference genome Wm82v4 (glyma.Wm82.gnm4), downloaded from SoyBase, using HISAT2 (v2.2.1), and the resulting SAM files were converted into sorted BAM files using SAMtools (v1.13). Gene annotation (.gff3) files were converted to GTF format using gffread, and gene-level read counts were obtained with featureCounts (v2.0.3) using the paired-end option. Differential expression analysis and normalization were conducted in R (4.4.1) using DESeq2 (v1.46.0), and DEGs were identified based on |log2(fold change)|≥ 2 and *Padj* < 0.01. Raw sequencing data have been deposited in ArrayExpress under the accession number E-MTAB-15662.

### KEGG and MapMan-based functional characterization of DEGs

DEGs were functionally annotated using the TB tools (v2.357) [36]. KEGG pathways were considered significantly enriched when the EF > 1 and the *p-*value ≤ 0.05. Enriched KEGG pathways were visualized using the ggplot2 package (v3.5.2) in R [37]. In addition, DEGs were assigned to functional categories using MapMan software (v.3.6.0RC1) with the Glycine max mapping file version X4.4 *Glycine max* [38]. KEGG pathway visualization was performed using The Bioconductor package pathview (v1.46.0). log₂ fold-change values of DEGs were overlaid on relevant KEGG pathway (gmx00940, gmx00941, gmx00943 and gmx00944). Pathview was run with the soybean organism code (gmx) and customized visualization parameters (limit = list(gene = max|log₂ fold-change|, cpd = 1)).

### Isoflavone extraction and quantification in root

Isoflavone standards, including daidzein, glycitein, genistein, daidzin, glycitin, genistin, malonyl-daidzin, malonyl-glycitin, malonyl-genistin, acetyl-daidzin, acetyl-glycitin, and acetyl-genistin, were purchased from Sigma-Aldrich (St. Louis, MO, USA). For extraction, the roots of Daewonkong and PI 471938 plants subjected to drought stress for 5 days were finely ground, and 1 g of powder was suspended in 20 mL of 50% methanol. The samples were stirred continuously at room temperature for 24 h. The extracts were centrifuged at 3,800 rpm for 10 min using a refrigerated centrifuge (Centrifuge 5810 R, Eppendorf, Hamburg, Germany) equipped with a Rotor S-4–104 and 50 mL conical tubes (SPL Life Sciences, Pocheon, Korea; SPL50150). The resulting supernatant was filtered through a 0.2 µm syringe filter (Millipore, Billerica, MA, USA), and the filtrates were transferred into 1.5 mL HPLC vials for further analysis.

Isoflavones were quantified using an HPLC system (Ultimate 3000, Dionex, Sunnyvale, CA, USA) equipped with a UV–Vis detector set at 260 nm. Separation was achieved on the Lichrospher RP-C18 column (5 µm, 4 × 125 mm; Merck, Darmstadt, Germany). The mobile phase consisted of solvent A (distilled water with 0.1% acetic acid) and solvent B (acetonitrile with 0.1% acetic acid) at a flow rate of 1.0 mL/min. The injection volume was 10 µL. Individual isoflavones were identified and quantified by comparing the retention times and UV spectra with those of authentic standards, as described by Dhungana et al. [39].

### Preparation of extracts for total polyphenol, ABTS, and DPPH assays

Roots of Daewonkong and PI 471938 subjected to drought stress for 5 days were oven-dried and ground into a fine powder, and 1 g of the dry weight (DW)-based powder was extracted with 20 mL of 80% ethanol in a shaking incubator (250 rpm) at room temperature for 24 h. The extracts were centrifuged at 3,800 rpm for 5 min, and the resulting supernatant was filtered through a 0.45 µm syringe filter (Millipore, Billerica, MA, USA) before analysis [39].

### Determination of total polyphenol content

Total polyphenol content was quantified using the Folin–Ciocalteu method, as described by Blainski et al. [40] with modifications. In a 96-well plate, 10 µL of root extracts were mixed with 100 µL of 10% Folin–Ciocalteu reagent and incubated for 5 min. Subsequently, 80 µL of 7.5% Na₂CO₃ was added, and the mixture was incubated in the dark for 30 min. A calibration curve was generated using gallic acid (0–500 ppm). Absorbance was measured at 750 nm using a spectrophotometer (Multiskan Spectrum, Thermo Fisher Scientific, Vantaa, Finland), and the results were expressed as GAE.

### ABTS radical scavenging activity

ABTS radical-scavenging activity was measured according to the method described by Lee and Cho [41], with slight modifications. ABTS stock solution was prepared by mixing 7.4 mM ABTS with 2.6 mM potassium persulfate (1:1, v/v) in ethanol and incubating the mixture in the dark at room temperature for 24 h. The solution was then diluted with ethanol to obtain an absorbance in the range of 0.2 to 1.0 at 735 nm. In a 96-well plate, 200 µL of the ABTS solution was mixed with 20 µL of root extracts or Trolox. After 30 min of incubation, absorbance was measured at 735 nm using the spectrophotometer described above. The scavenging activity was expressed as TE.

### DPPH radical scavenging activity

DPPH radical scavenging activity was determined following the method of Cho et al. [42]. In a 96-well plate, 20 µL of root extracts or Trolox was added to 200 µL of 0.2 mM DPPH solution. The mixture was incubated in the dark at room temperature for 30 min and the absorbance was measured at 520 nm using the same spectrophotometer. The results were expressed as TE.

### RNA isolation, cDNA synthesis, and qRT-PCR for gene expression analysis

Root samples (as described above) were immediately frozen in liquid nitrogen and stored at − 80 °C until use. Total RNA was extracted using the RNeasy Plant Mini Kit (Qiagen, Hilden, Germany) according to the manufacturer’s instructions. First-strand cDNA was synthesized from 1 µg of total RNA using M-MuLV Reverse Transcriptase (Thermo Fisher Scientific, Waltham, MA, USA) and an oligo(dT)18 primer. qRT-PCR was performed using a QuantStudio 5 Real-Time PCR System (Thermo Fisher Scientific) with SYBR Green detection chemistry [43]. The soybean Actin11 gene (*Act11/Glyma.18G290800*) was used as an internal reference [44]. Statistical analysis was performed using Student’s t-test, and significance was determined at *p* < 0.05. The primer sequences are listed in Additional file 4: Table S4.

## Supplementary Information

Additional file 1: Table S1. List of DEGs identified in Daewonkong roots under drought stress compared to the control.

Additional file 2: Table S2. KEGG enrichment results for upregulated and downregulated genes under drought stress.

Additional file 3: Table S3. Summary of raw and trimmed RNA-seq data statistics.

Additional file 4: Table S4. Primer sequences used for qRT-PCR analysis of drought-responsive genes in soybeans.

Additional file 5: Table S5. Detailed MapMan term assignments for genes upregulated and downregulated under drought stress.

Additional file 6: Figure S1. Illustration of DEGs in phenylpropanoid and isoflavonoid biosynthesis pathway

## Data Availability

Raw data were uploaded to the ArrayExpress public repository (accession number: E-MTAB-15662).

## Abbreviations

RNA-seq: RNA sequencing
KEGG: Kyoto Encyclopedia of Genes and Genomes
HPLC: High-performance liquid chromatography
ROS: Reactive oxygen species
TCA: Tricarboxylic acid
MAPK: Mitogen-activated protein kinase
NBS-LRR: Nucleotide-binding site-leucine-rich repeat
DAD: Diode array detection
DEG: Differentially expressed genes
ABTS: 2,2′-Azino-bis(3-ethylbenzothiazoline-6-sulfonic acid)
DPPH: 2,2-Diphenyl-1-picrylhydrazy
TE: Trolox equivalents
qRT-PCR: Quantitative real-time PCR
