# Supplementary figures and images for: Unravelling mechanisms of drought tolerance in a soybean cultivar (Daewonkong roots): insights into integrative transcriptomic and metabolite analyses
Source: BMC Plant Biol. 2026 Jan 15;26:274. doi: 10.1186/s12870-026-08144-2 (PMC12892709; doi:10.1186/s12870-026-08144-2)

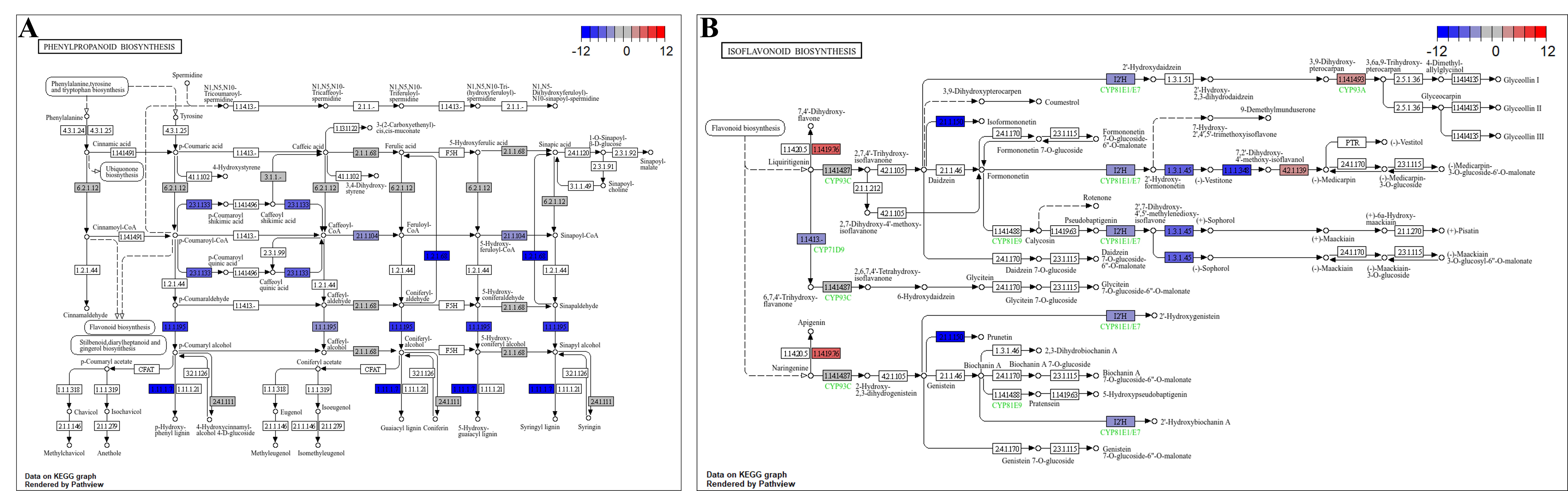

Supplement: Supplementary file 6 — Additional file 6: Figure S1. Illustration of DEGs in phenylpropanoid and isoflavonoid biosynthesis pathway [file 12870_2026_8144_MOESM6_ESM.jpg]
